# Metabolites Associated with the Main Nutrients in Two Varieties of Purple Rice Processed to Polished Rice

**DOI:** 10.3390/metabo13010007

**Published:** 2022-12-20

**Authors:** Qiangqiang Xiong, Runnan Wang, Changhui Sun, Ruizhi Wang, Xiaoyu Wang, Yu Zhang, Hongcheng Zhang, Jinyan Zhu

**Affiliations:** 1 Jiangsu Key Laboratory of Crop Genetics and Physiology/Jiangsu Key Laboratory of Crop Cultivation and Physiology, Agricultural College of Yangzhou University, Yangzhou 225009, China; 2 Jiangsu Co-Innovation Center for Modern Production Technology of Grain Crops, Yangzhou University, Yangzhou 225009, China

**Keywords:** purple rice, amino acid, mineral elements, nutrients, citric acid

## Abstract

Under the same nitrogen fertilizer and cultivation conditions, the nutrients of rice are strongly affected during the processing of brown rice to polished rice, especially in special rice varieties. In this study, twenty-two amino acids in brown and polished rice of two purple rice varieties were quantified using targeted metabolomics, and the relationships among the main nutrients, mineral elements and metabolites were analysed. The results showed that the amino acid levels in YZN1_H (polished rice of Yangzinuo No. 1) and YZN2_H (polished rice of Yangzinuo No. 2) decreased to different degrees compared with those in YZN1_B (brown rice of Yangzinuo No. 1) and YZN2_B (brown rice of Yangzinuo No. 2). Citric acid is closely associated with amino acids. The total sugar (TS), ATP, and soluble dietary fiber (SDF) levels in YZN1_B decreased by 9.37%, 53.85%, and 75.71%, respectively, compared with those in YZN1_H. The TS, ATP, and SDF levels in YZN2_B decreased significantly by 6.92%, 21.03%, and 76.78%, respectively, compared with those in YZN2_H. Citric acid was significantly negatively correlated with ATP and SDF but significantly positively correlated with carotenoids. The Se content in YZN1_H was significantly higher than that in YZN1_B by 87.02%. The Se content in YZN2_H was significantly higher than that in YZN2_B by 72.02%. Citric acid was significantly positively correlated with Fe, Mn, Ca, and Mg. Citric acid was identified as a candidate key metabolite that affects changes in the main nutrients in purple rice during processing.

## 1. Introduction

Rice is one of the most important food crops in the world. As the largest rice producer in the world, China is rich in rice varieties, including special rice varieties [1]. Rice grains are composed of two parts, namely the husk and brown rice. Brown rice is composed of the pericarp, seed coat, aleurone layer, endosperm, and embryo [2]. The nutritional structure of rice is closely related to human health [3]. With the rapid development of China’s economy and the continuous improvement in people’s living standards, urban and rural residents have an increasingly strong need for high-quality rice for nutritional and health benefits [4]. However, for decades, rice breeding in China has been focused on improvements in rice yield while ignoring research and development on high-quality and functional rice, resulting in mainly a single type of rice being available in the rice market [1]. With the rapid development of the modern rice processing industry and the need to adjust the crop planting structure, the demand for special rice varieties is increasing annually [5]. Rice farmers have a strong desire to achieve higher agricultural incomes by planting special high-quality and high-nutrition rice varieties. The development of advanced, diverse, and functional rice varieties will inevitably lead to further social development [6,7]. Bioaugmentation of rice nutrients generally refers to improving the utilization of trace elements in food crops by biological breeding, genetic engineering, or agricultural measures involving fertilizer application [8]. The application of foliar zinc fertilizer can improve the zinc nutrition level of rice grains to varying degrees [9]. Eight key genes related to anthocyanin synthesis were transferred into rice through genetic engineering for the specific synthesis of anthocyanins in the rice endosperm, leading to the creation of a new rice germplasm, “amethyst rice”, which is rich in anthocyanins [10]. The development of special rice varieties with high levels of lysine, trace elements, vitamins, and other nutrients, as well as flavonoids, alkaloids, anthocyanins, and other physiologically active substances, is important [11,12,13]. Active research will promote the rapid development of breeding strategies for such special rice varieties, which will play an important role in improving people’s dietary health, promoting agricultural development, and increasing farmers’ income. In addition, with the refinement and diversification of consumers’ dietary structures, rice processing methods are also being further improved, resulting in the loss of protein, fat, and mineral elements on the surface of rice during processing [5,14]. This is especially true for the processing of specialty rice varieties. In summary, the research shows that during the process of grinding brown rice to polished rice, the organizational structure and composition of rice undergo significant changes.

With continuous development in the field of biotechnology, liquid chromatography–mass spectrometry (LC-MS)-based metabolomic technology is being increasingly studied for the detection of changes in the types and quantities of metabolite molecules [14,15]. Metabolites are substances produced or consumed by metabolic processes. Biomacromolecules are not included, as their precursors and degradation products are real metabolites. Our team used metabolomic technology in a previous study and found that the total antioxidant capacity of purple rice was significantly higher than that of white rice, and flavonoids and phenol metabolites were closely related to the total antioxidant capacity [11]. Then, the metabolites related to nutrients in purple rice were further analyzed, and the regulatory roles of key metabolites in metabolic pathways were elucidated [12]. However, the effect of processing on the nutritional and metabolic components of purple rice warrants further study. In this study, Yangzinuo No. 1 and Yangzinuo No. 2 were used as experimental materials to explore the changes in the main nutrient components and mineral elements and their relationships with the metabolites of polished rice derived by processing brown rice. This study provides a theoretical basis for the cultivation and processing of special functional rice varieties with excellent nutritional quality.

## 2. Materials and Methods

### 2.1. Plant Materials and Sample Collection

Yangzinuo No. 1 (YZN1) and Yangzinuo No. 2 (YZN2) were independently cultivated by the Agricultural College of Yangzhou University. Two purple rice varieties were planted at the Shatou base of Yangzhou University. The rice sowing date was 25 May 2021. The blanket seedlings were grown, with four seedlings planted in each hole. Each variety was planted in three replicates. Two purple rice varieties were planted in high-standard farmland with good drainage and irrigation conditions. Compound fertilizer was used for rice fertilization, with nitrogen, phosphorus, and potassium accounting for 15% of the total composition. A total of 360 kg hm^−2^ of pure nitrogen was applied. The base fertilizer:tiller fertilizer:panicle fertilizer ratio was 5:3:2. When the plants of the two purple rice varieties had matured, the rice was milled separately as brown rice and polished rice (one polishing). The grain length of YZN1 is 6.43 mm, and the width is 3.61 mm. The grain length of YZN2 is 6.56 mm, and the width is 3.64 mm. The brown rice and polished rice grains of Yangzinuo No. 1 were designated YZN1_B and YZN1_H, respectively. The brown rice and polished rice grains of Yangzinuo No. 2 were designated YZN2_B and YZN2_H, respectively. Three biological replicates from both purple rice varieties were sampled. All samples were flash frozen in liquid N and stored at −80 °C until measurements were performed.

### 2.2. Determination of Crude Fat, Crude Protein, Total Sugar, and Mineral Element Levels

The amino acid content was determined according to the method of Xiong et al. (2022) [12]. The standard curve of amino acids is shown in Table S1. Determination of the crude fat (CF) content was performed according to the National Standards of the People’s Republic of China (NSPRC) (GB 5009.6-2016) [16]. Determination of the crude protein (CP) content was performed according to NSPRC (GB 5009.5-2016) [17]. The carotenoid (CAROT) content [18] and total sugar (TS) content (NSPRC GB/T 15672-2009) [19] were determined by Suzhou Michy Biomedical Technology Co., Ltd. (Suzhou Michy Biomedical Technology Co., Ltd., Suzhou, China). Weigh 0.1200 g of rice grain sample and add it into a polytetrafluoroethylene digestion tank, then add 5 mL of concentrated nitric acid and 2 mL of 30% hydrogen peroxide. Digest until the mixture is clear, cool it down, fix the volume to 25 mL, and filter it (the content of selenium element is determined by atomic fluorescence meter). The plant element content was determined by inductively coupled plasma—mass spectrometry/atomic emission spectrometry (ICP-MS/AES).

### 2.3. Determination of the ATP Content

A 0.2000 g sample of rice grain was added to 1.5 mL of 0.6 M perchloric acid solution, subjected to ultrasonic extraction for 1 h, and centrifuged at 8000× *g* for 10 min. One millilitre of the supernatant was taken, adjusted to neutral pH with NaOH solution, and brought up to 1.5 mL with water. The samples were filtered and tested.

The liquid chromatography analysis conditions were as follows: chromatograph, Agilent 1100 high-performance liquid chromatograph with a wavelength of 254 nm (Santa Clara, CA, USA); chromatographic column, Kromasil C18-BP reversed-phase column (250 mm × 4.6 mm, 5 μm); column temperature, 30 °C; flow rate, 1 mL min^−1^; injection volume, 10 μL; mobile phase, buffer matrix (0.1 mol NaH_2_PO_4_ solution and 0.1 mol NaH_2_PO_4_ solution adjusted to pH 7.0).

The standard curve was determined as follows. The ATP standard was accurately weighed and dissolved in water to obtain standard solutions of 1, 4, 10, 40, 100, and 200 μg mL^−1^. The peak area of each standard solution was then determined according to the above chromatographic conditions. With this value on the ordinate and the concentration on the abscissa, the standard curve of ATP was generated, and within the linear range, the correlation coefficient was calculated (Table S2).

### 2.4. Determination of the Soluble Dietary Fiber Content

A total of 1.0000 g of the sample was accurately weighed and placed in a high-footed bottle for determination of soluble dietary fiber (SDF) content. Then, 50 mL of MES-TRIS buffer was added, and the sample was stirred until completely dispersed. Then, 100 μL of high-temperature-resistant α-amylase was added, and the sample was covered with aluminum foil and stirred at low speed in a water bath at 80 °C for 30 min. The tall bottle was removed and cooled to 60 °C, and a spatula was used to remove the mesh on the edge of the tall bottle and the glue at the bottom. The sides of the bottle were scraped to allow complete enzymatic hydrolysis of the sample, and the tall flask and spatula were rinsed with distilled water. The pH was adjusted to 7.5 ± 0.2 with 0.275 mol L^−1^ NaOH. Then, 100 μL of protease solution was added, and the sample was covered with aluminum foil and continuously shaken for 30 min in a 60 °C water bath.

The filtrate produced by suction filtration of insoluble dietary fiber was collected into a preweighed 600 mL high-footed beaker, and the filtrate volume was estimated by weighing the total mass of the beaker with filtrate and deducting the mass of the beaker. Four times the volume of 95% ethanol preheated to 60 °C was added to the filtrate, and the sample was allowed to precipitate at room temperature for 1 h. Then, diatomaceous earth was wetted with 15 mL of 98% ethanol, redistributed in a preweighed crucible, and suctioned to form a flat filter layer of diatomaceous earth. Next, 78% ethanol and a spatula were used to transfer all the contents of the high-foot flask to the crucible, and the residue was rinsed twice with 15 mL of 78% ethanol, 95% ethanol, and acetone. The crucible was dried at 105 °C for 5 h, placed in a desiccator to cool for 0.5 h, and weighed to an accuracy of 0.1 mg. The weights of the empty crucible and diatomaceous earth were subtracted to obtain the weight of the residue. One of the two sample residues was used to measure the nitrogen (N) content, and 6.25 was used as the conversion factor to calculate the protein mass (mP). The ash content of the other sample was measured as follows: ashing at 525 °C for 5 h, cooling in a desiccator, accurately weighing the total mass of the crucible (accurate to 0.1 mg), subtracting the mass of the crucible after treatment, and calculating the ash mass (mA).

(1) $$\mathrm{SDF}\;\mathrm{content}\;\frac{mR-mP-mA}{m}\;\times \;100$$

where ***mR*** is the average residue mass of the duplicate samples (in grams); ***mP*** is the mass of protein in the sample residue (in grams); ***mA*** is the mass of ash in the sample residue (in grams); and ***m*** is the mean sampling mass of the duplicate samples (in grams).

### 2.5. Statistical Analysis

Adobe Illustrator CS6 and WPS2021 were used to arrange and map the physiological and biochemical data. SPSS 18.0 software was used for correlation analysis and significance analysis (Tukey’s test).

## 3. Results

### 3.1. Analysis of Amino Acid Content

Twenty-two amino acids were detected, and the total ion chromatograms (TICs) showed that each index had high resolution and good peak shape (Figure S1). The linear R2 of all amino acid indicators was greater than 0.99, indicating good linearity (Table S1). The relative standard deviation of each target was less than 15% (Table S1), which indicated that the method and analysis system were stable and reliable and could be used for the quantitative detection of samples. The content of glycine, cysteine, hydroxyproline, methyl, and serine in grain samples is lower than the limit of quantification (LOQ), but within the limit of detection (LOD) (Table S1).

Yangzinuo No. 1 and Yangzinuo No. 2 showed different degrees of amino acid loss during the processing of brown rice to polished rice (Table 1). The levels of Ala, Arg, Asn, Asp, Glu, Gly, His, Ile, Leu, Hyp, Trp, Lys, Met, Phe, Pro, Ser, Thr, Tyr, and Val in YZN1_H were significantly lower than those in YZN1_B by 75.41%, 75.63%, 50.19%, 60.63%, 61.3%, 61.42%, 65.82%, 84.81%, 83.87%, 68.27%, 32%, 76.39%, 72%, 85.07%, 65.54%, 54.3%, 71.74%, 82.96%, and 79.75%, respectively. The levels of Ala, Arg, Asn, Asp, Gln, Glu, Gly, His, Cys, Leu, Hyd, Trp, Lys, Phe, Ser, Thr, and Val in YZN2_H were significantly lower than those in YZN2_B by 63.3%, 64.15%, 43.66%, 51.87%, 71.08%, 40.07%, 40.82%, 59.52%, 55.14%, 55.74%, 46.08%, 30.77%, 47.95%, 57.35%, 61.24%, 57.4%, and 81.7%, respectively. In addition, the levels of Ala, Asn, Asp, Gly, and Cys in YZN2_B were significantly higher than those in YZN1_B. The levels of Asn, Asp, Gly, Cys, and Met in YZN2_H were significantly higher than those in YZN1_H.

### 3.2. Analysis of the Main Nutrients

The levels of the main nutrients in Yangzinuo No. 1 and Yangzinuo No. 2 changed to different degrees during the processing of brown rice to polished rice (Table 2). Compared with those in YZN1_B, the CAROT, CF, and CP levels in YZN1_H decreased significantly by 43.15%, 77.63%, and 8.96%, respectively. Compared with those in YZN2_B, the CAROT, CF, and CP levels in YZN2_H decreased significantly by 64.88%, 61.65%, and 9.21%, respectively. Compared with those in YZN1_H, the TS, ATP, and SDF levels in YZN1_B decreased significantly by 9.37%, 53.85%, and 75.71%, respectively. Compared with those in YZN2_H, the TS, ATP, and SDF levels in YZN2_B decreased significantly by 6.92%, 21.03%, and 76.78%, respectively.

### 3.3. Analysis of Seven Nutrient Elements

In addition to differences in the Se content, Yangzinuo No. 1 and Yangzinuo No. 2 showed different degrees of loss of nutrient elements during the processing of brown rice to polished rice (Table 3). Compared with the levels in YZN1_B, the Fe, Mn, Zn, Ca, and Mg levels in YZN1_H decreased significantly by 39.47%, 58.86%, 24.37%, 36.44%, and 64.66%, respectively. Compared with the levels in YZN2_B, the Fe, Mn, Zn, Ca, and Mg levels in YZN2_H decreased significantly by 33.58%, 61.68%, 23.99%, 30.46%, and 62.26%, respectively. The Cu levels in YZN1_B and YZN2_B were 5.56% and 14.47% higher than those in YZN1_H and YZN2_H, respectively, and these differences were not significant. The Se content in YZN1_H was 87.02% higher than that in YZN1_B. The Se content in YZN2_H was significantly higher than that in YZN2_B by 72.02%. The Se content was very low in the two purple rice varieties.

### 3.4. Correlation Analysis of the Amino Acids and Metabolites

**Figure 1:**
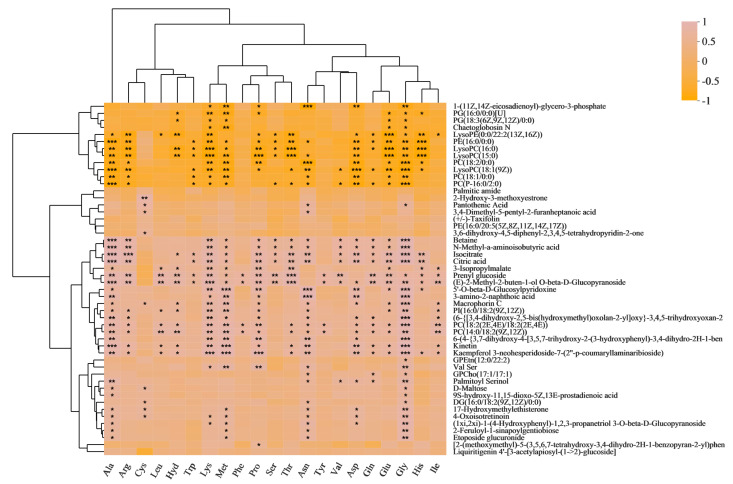
Correlation analysis of amino acids with differential metabolites (DMs). Each grid represents the correlation between the two attributes, and different colors represent the sizes of the correlation coefficients between the attributes (the same applies below). * *p* < 0.05; ** *p* < 0.01; *** *p* < 0.001.

Based on our previously published data [20], we wished to understand the relationships between amino acids and metabolites during the processing of purple rice from brown rice to polished rice. We conducted a correlation analysis between twenty-two amino acids and differential metabolites (Figure 1). Chaetoglobosin N was significantly negatively correlated with Lys, Met, Glu, and Gly. Pantothenic acid and 3,4-dimethyl-5-pentyl-2-furanheptanoic acid were significantly positively correlated with Cys and Asn. Betaine, N-methyl-a-aminoisobutyric acid, isocitrate, and citric acid were significantly positively correlated with Ala, Arg, Lys, Met, Pro, Ser, Thr, Asn, Val, Asp, Gln, Glu, and Gly. 3-Isopropylmalate and prenyl glucoside were significantly positively correlated with Ala, Leu, Hyd, Lys, Met, Pro, Thr, Glu, His, and Ile. Macrophorin C and kinetin were significantly positively correlated with Ala, Hyd, Lys, Met, Pro, Asn, Asp, Glu, Gly, and Ile. Palmitoyl serinol, D-maltose, and etoposide glucuronide were significantly positively correlated with Ala and Gly.

### 3.5. Correlation Analysis of the Main Nutrients and Metabolites

**Figure 2:**
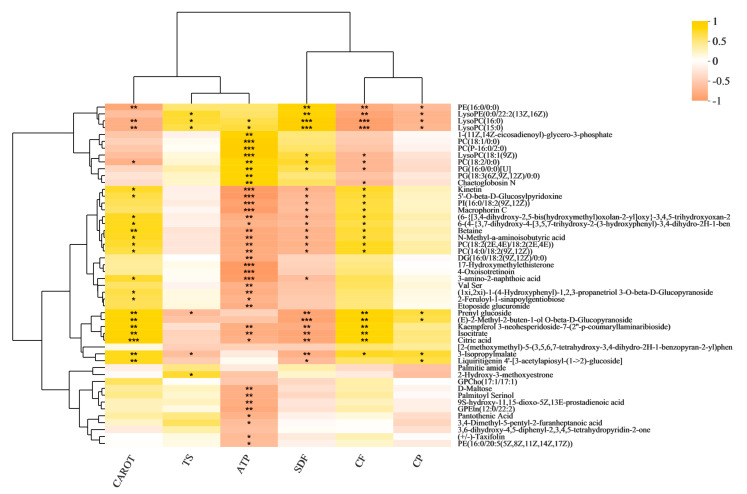
Correlation analysis of the major nutrients with DMs. * *p* < 0.05; ** *p* < 0.01; *** *p* < 0.001.

Based on our previously published data [20], we wished to understand the relationships between the main nutrient indexes and metabolites during the processing of purple rice from brown rice to polished rice. We conducted a correlation analysis among the CAROT, TS, ATP, SDF, CF, CP, and differential metabolite levels (Figure 2). Chaetoglobosin N was significantly negatively correlated with CF. Chaetoglobosin N was significantly positively correlated with ATP. Kinetin, betaine, N-methyl-a-aminoisobutyric acid, isocitrate, and citric acid were significantly negatively correlated with ATP and SDF. Kinetin, betaine, N-methyl-a-aminoisobutyric acid, isocitrate, and citric acid were significantly positively correlated with CAROT and CF. Etoposide glucuronide was significantly negatively correlated with ATP. D-Maltose, palmitoyl serinol, pantothenic acid, and taxifolin were significantly negatively correlated with ATP. 3-Isopropylmalate was significantly negatively correlated with TS and SDF. 3-Isopropylmalate was significantly positively correlated with CAROT, CF, and CP.

### 3.6. Correlation Analysis of Seven Nutrients and Metabolites

**Figure 3:**
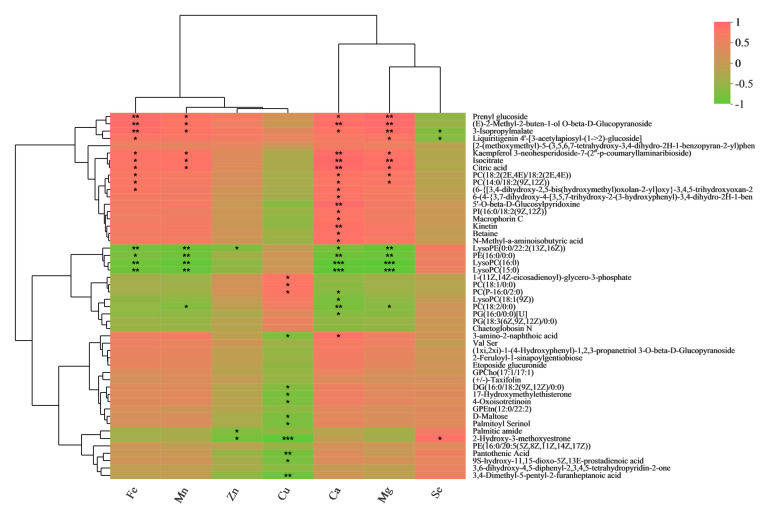
Correlation analysis of Fe, Mn, Zn, Cu, Ca, Mg, and Se with DMs. * *p* < 0.05; ** *p* < 0.01; *** *p* < 0.001.

**Figure 4:**
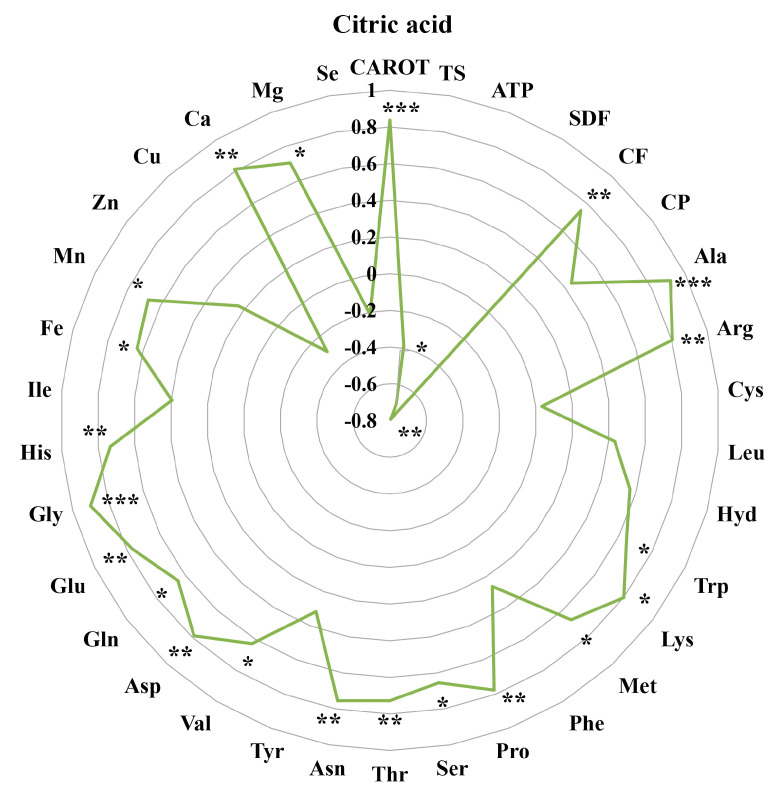
Correlation analysis between citric acid and the main nutrients and mineral elements. * *p* < 0.05; ** *p* < 0.01; *** *p* < 0.001.

Based on our previously published data [20], we wished to understand the relationships between seven nutrients and metabolites during the processing of purple rice from brown rice to polished rice. We carried out a correlation analysis of Fe, Mn, Zn, Cu, Ca, Mg, and Se with differential metabolites (Figure 3 and Figure 4). Prenyl glucoside, 3-isopropylmalate, isocitrate, and citric acid were significantly positively correlated with Fe, Mn, Ca, and Mg. Macrophorin C, kinetin, betaine, and N-methyl-a-aminoisobutyric acid were significantly positively correlated with Ca. D-Maltose, palmitoyl serinol, and pantothenic acid were significantly negatively correlated with Cu. Palmitic amide was significantly negatively correlated with Zn. 3-Isopropylmalate was significantly negatively correlated with Se.

## 4. Discussion

**Figure 5:**
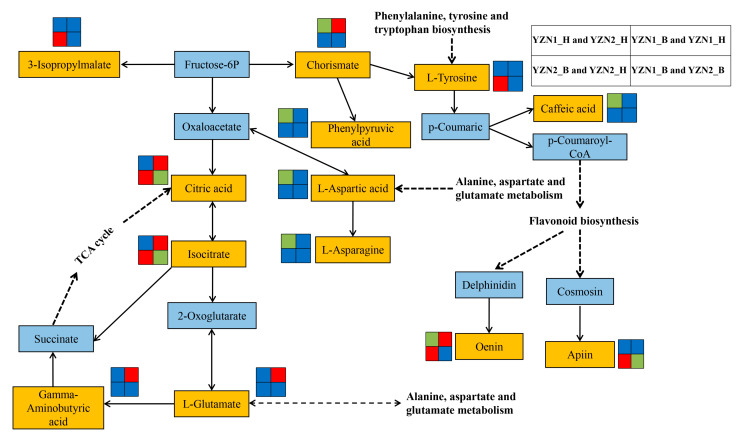
Overview of the possible regulatory network of some key metabolites in metabolic pathways in pairwise comparisons of two rice varieties before and after processing. The key metabolites are shown in the orange rectangles. Small red rectangles indicate significantly upregulated metabolites; small green rectangles indicate significantly downregulated metabolites; and small blue rectangles indicate no significant difference in metabolites.

The levels of various amino acids, especially essential amino acids, determine the nutritional value of rice protein, especially the lysine content, which is a major indicator of the nutritional quality of rice [21,22]. In this study, there was no significant difference in the Lys content between YZN1_B and YZN2_B (Table 1). During rice processing, the nutritional content of rice is mainly affected by human factors [23]. The Lys content in the two purple rice cultivars decreased significantly during processing from brown rice to polished rice (Table 1). During the processing of brown rice to polished rice, most of the amino acid content is lost at varying degrees, and nutrients are lost along with the byproducts of processing. The quantity and variety of metabolites (including nutrients) of the two white rice varieties decreased significantly during the process of grain processing to milled rice; lipids and flavonoids may be important substances that lead to differences in taste and nutritional quality between the two varieties [24]. Purple rice is rich in macronutrients, such as protein, fat, and carotenoids, as well as micronutrients, such as vitamins, and phytosterols, that are beneficial to human health. Long-term consumption of purple rice can reduce the incidence of chronic diseases, such as coronary heart disease, diabetes, and obesity [1]. Carotenoid is a general term for a class of natural pigments that are widely present in animals, higher plants, and fungi. Carotenoids have antioxidant, immunomodulatory, anticancer, and antiaging effects [25]. In this study, the CAROT levels in YZN1_B and YZN2_B were significantly higher than those in YZN1_H and YZN2_H (Table 2). This was consistent with the conclusion that the CAROT levels in the brown rice of the two purple rice cultivars were higher than those in the polished rice of these cultivars. Citric acid has chelating and pH-adjusting properties, making it possible to increase antioxidant activity, inhibit enzyme activity, and prolong the shelf life of processed quick-frozen foods [26,27]. Citric acid was significantly positively correlated with CAROT (Figure 2 and Figure 4). Dietary fiber can promote intestinal peristalsis and prevent obesity, cardiovascular disease, and constipation [28]. In this study, the SDF levels in YZN1_H and YZN2_H were significantly higher than those in YZN1_B and YZN2_B (Table 2). Therefore, the consumption of processed milled rice from purple rice may be beneficial for individuals with insufficient gastrointestinal motility. Carbohydrates, fats, and proteins are the three major nutrients in humans and animals [29]. Carbohydrates are mainly oxidatively decomposed to provide the energy required for life activities. Fat is mainly resynthesized in the body and stored. Proteins are digested and decomposed to amino acids, and these amino acids are mainly used to synthesize various tissue proteins, enzymes, and certain hormones in organisms [30]. In this study, the TS levels in YZN1_H and YZN2_H were significantly higher than those in YZN1_B and YZN2_B (Table 2). ATP is the most direct source of energy in living organisms. In this study, the ATP levels in YZN1_H and YZN2_H were higher than those in YZN1_B and YZN2_B (Table 2). Taxifolin is a novel food antioxidant that, similar to other flavonoids, has a variety of biological activities in the human body, including antioxidant, free radical scavenging, antiviral, and antivasodilation activities [31]. Citric acid and taxifolin were significantly negatively correlated with ATP (Figure 2 and Figure 4). After purple rice is processed to polished rice, the nutrients are mainly distributed in the aleurone layer, resulting in an increase in the TS content in the polished rice. The CF and CP levels in YZN1_B and YZN2_B were significantly higher than those in YZN1_H and YZN2_H (Table 2). Mineral elements are necessary for normal physiological activities of the human body. They cannot be synthesized in the human body and must be ingested via food. Excessive or insufficient intake can cause some diseases [32,33]. Clinical trials have confirmed that black rice and red rice can lower blood lipid levels and reduce the risk of arteriosclerosis [34]. The Fe contained in special rice varieties can alleviate nutritional anemia [35]. Zn can improve immunity and prevent skin roughness and dampness [36]. In this study, the Fe, Mn, Zn, Ca, and Mg levels in YZN1_H were significantly lower than those in YZN1_B (Table 3). The Fe, Mn, Zn, Ca, and Mg levels in YZN2_H were significantly lower than those in YZN2_B (Table 3). This result indicated that the levels of mineral elements in the brown rice of the two purple rice cultivars were high, except for Se. The Se content was relatively low in the two purple rice varieties. The Se content in YZN1_H was significantly higher than that in YZN1_B. The Se content in YZN2_H was significantly higher than that in YZN2_B. Citric acid has been shown to have antioxidant and cholesterol-lowering functions [37,38]. Citric acid was significantly positively correlated with Fe, Mn, Ca, and Mg (Figure 3 and Figure 4). In this study, citric acid was identified as a candidate metabolic marker during the processing of purple rice. In addition, the metabolite isocitrate was also identified as a candidate metabolic marker during this rice processing step. Citric acid and isocitrate are also key metabolites in metabolic pathways and play important regulatory roles (Figure 5). This finding provides a theoretical basis for adding nutritional value during food processing and metabolomics-assisted breeding.

## 5. Conclusions

In this study, the changes in the levels of twenty-two amino acids, CF, CP, TS, CAROT, ATP, SDF, and seven mineral elements in two purple rice varieties processed from brown rice to polished rice were detected. Yangzinuo No. 1 and Yangzinuo No. 2 showed different degrees of amino acid content loss during the processing of brown rice to polished rice. The levels of CAROT, CF, and CP in YZN1_H were significantly lower than those in YZN1_B. The CAROT, CF, and CP levels in YZN2_H were significantly lower than those in YZN2_B. The levels of TS, ATP, and SDF in YZN1_B were significantly lower than those in YZN1_H. The levels of TS, ATP, and SDF in YZN2_B were significantly lower than those in YZN2_H. The levels of CAROT, CF, and CP in the polished rice of the two purple rice cultivars were significantly lower than those of the brown rice. The Fe, Mn, Zn, Ca, and Mg levels in the polished rice of the two purple rice varieties were significantly lower than those in the brown rice. However, the levels of TS, ATP, and SDF in the polished rice of the two purple rice cultivars were significantly higher than those in the brown rice. Citric acid was significantly positively correlated with Ala, Arg, Lys, Met, Pro, Ser, Thr, Asn, Val, Asp, Gln, Glu, and Gly but significantly negatively correlated with ATP and SDF. Citric acid was significantly positively correlated with CAROT, while it was significantly positively correlated with Fe, Mn, Ca, and Mg. Citric acid was thus identified as a key metabolite in the processing of purple rice. The results of this study provide a theoretical basis for metabolomics-assisted breeding.

## Figures and Tables

**Table 1:** Difference analysis of the amino acid content in brown rice grains of two purple rice varieties after processing to polished rice.

Amino Acid Name	YZN1_H	YZN1_B	YZN2_H	YZN2_B
L-alanine (Ala) (ng mg^−1^)	1.21 c	4.92 b	3.49 bc	9.51 a
L-arginine (Arg) (ng mg^−1^)	1.07 b	4.39 a	1.90 b	5.30 a
L-asparagine anhydrous (Asn) (ng mg^−1^)	5.22 c	10.48 b	15.91 b	28.24 a
L-aspartic acid (Asp) (ng mg^−1^)	5.26 c	13.36 b	12.22 b	25.39 a
L-cystine (Cys-Cys) (ng mg^−1^)	0.00	0.00	0.00	0.00
L-glutamine (Gln) (ng mg^−1^)	1.05 b	2.37 ab	0.94 b	3.25 a
L-glutamic acid (Glu) (ng mg^−1^)	8.65 b	22.35 a	13.16 b	21.96 a
Glycine (Gly) (ng mg^−1^)	0.49 c	1.27 b	1.16 b	1.96 a
L-histidine (His) (ng mg^−1^)	0.27 b	0.79 a	0.34 b	0.84 a
L-isoleucine (Ile) (ng mg^−1^)	0.12 b	0.79 a	0.30 b	0.49 ab
L-cysteine (Cys) (ng mg^−1^)	0.0035 c	0.0077 c	0.0157 b	0.035 a
L-leucine (Leu) (ng mg^−1^)	0.15 c	0.93 a	0.27 c	0.61 b
L-hydroxyproline (Hyp) (ng mg^−1^)	0.0481 b	0.1516 a	0.0646 b	0.1198 a
L-tryptophan (Trp) (ng mg^−1^)	0.68 b	1.00 a	0.90 b	1.30 a
L-lysine (Lys) (ng mg^−1^)	0.17 b	0.72 a	0.38 b	0.73 a
L-methionine (Met) (ng mg^−1^)	0.07 b	0.25 a	0.20 a	0.26 a
L-phenylalanine (Phe) (ng mg^−1^)	0.20 c	1.34 a	0.29 c	0.68 b
L-proline (Pro) (ng mg^−1^)	0.51 b	1.48 a	0.91 ab	1.66 a
L-serine (Ser) (ng mg^−1^)	2.18 b	4.77 a	2.06 b	5.34 a
L-threonine (Thr) (ng mg^−1^)	0.52 b	1.84 a	0.72 b	1.69 a
L-tyrosine (Tyr) (ng mg^−1^)	0.38 b	2.23 a	0.53 b	1.16 ab
L-valine (Val) (ng mg^−1^)	0.49 b	2.42 a	0.73 b	3.99 a

Different lowercase letters represent significance at the 0.05 level (the same applies below). *n* = 3.

**Table 2:** Difference analysis of the nutrient levels in brown rice of two purple rice varieties after processing to polished rice.

Name	YZN1_H	YZN1_B	YZN2_H	YZN2_B
CAROT (μg g^−1^ FW)	4.77 c	8.39 b	4.32 c	12.30 a
TS (mg g^−1^ FW)	82.03 ab	74.34 c	85.56 a	79.64 b
ATP (μg g^−1^ FW)	40.15 a	18.53 b	8.37 c	6.61 c
SDF (g g^−1^)	4.57% a	1.11% c	4.22% b	0.98% c
CF (g g^−1^)	0.49% d	2.19% a	0.79% c	2.06% b
CP (g kg^−1^)	93.00 c	102.15 a	88.79 d	97.80 b

Different lowercase letters represent significance at the 0.05 level (the same applies below). *n* = 3.

**Table 3:** Difference analysis of the nutrient levels in brown rice of two purple rice varieties after processing to polished rice.

Name	YZN1_H	YZN1_B	YZN2_H	YZN2_B	LOD (mg L^−1^)	LOQ (mg L^−1^)
Fe (mg kg^−1^)	19.05 b	31.47 a	19.21 b	28.92 a	0.001	0.003
Mn (mg kg^−1^)	8.36 b	20.32 a	8.02 b	20.93 a	0.002	0.007
Zn (mg kg^−1^)	11.02 c	14.57 a	8.81 d	11.59 b	0.0001	0.0003
Cu (mg kg^−1^)	3.42 a	3.61 a	2.28 b	2.61 b	0.0001	0.0003
Ca (mg kg^−1^)	100.87 b	158.69 a	119.78 b	172.25 a	0.002	0.007
Mg (g kg^−1^)	0.41 c	1.16 a	0.40 c	1.06 b	0.002	0.007
Se (mg kg^−1^)	0.0533 b	0.0285 c	0.0836 a	0.0486 b	0.001	0.003

Different lowercase letters represent significance at the 0.05 level (the same applies below). LOD, limit of detection; LOQ, limit of quantification; *n* = 3.

## Data Availability

Not applicable.

## Supplementary Materials

The following supporting information can be downloaded at https://www.mdpi.com/article/10.3390/metabo13010007/s1, Figure S1: Total ion chromatogram (TIC) plots of the twelve samples. (A) YZN1_B1; (B) YZN1_B2; (C) YZN1_B3; (D) YZN1_H1; (E) YZN1_H2; (F) YZN1_H3; (G) YZN2_B1; (H) YZN2_B2; (I) YZN2_B3; (J) YZN2_H1; (K) YZN2_H2; and (L) YZN2_H3. Table S1: Linear equations for the twenty-two amino acids; Table S2: Linear equations for ATP.
