# Supplementary material for: Metabolites Associated with the Main Nutrients in Two Varieties of Purple Rice Processed to Polished Rice
Source: Metabolites. 2022 Dec 20;13(1):7. doi: 10.3390/metabo13010007 (PMC9867293; doi:10.3390/metabo13010007)
Supplement: Supplementary file 1 [file metabolites-13-00007-s001.zip › Figure S1.pdf]

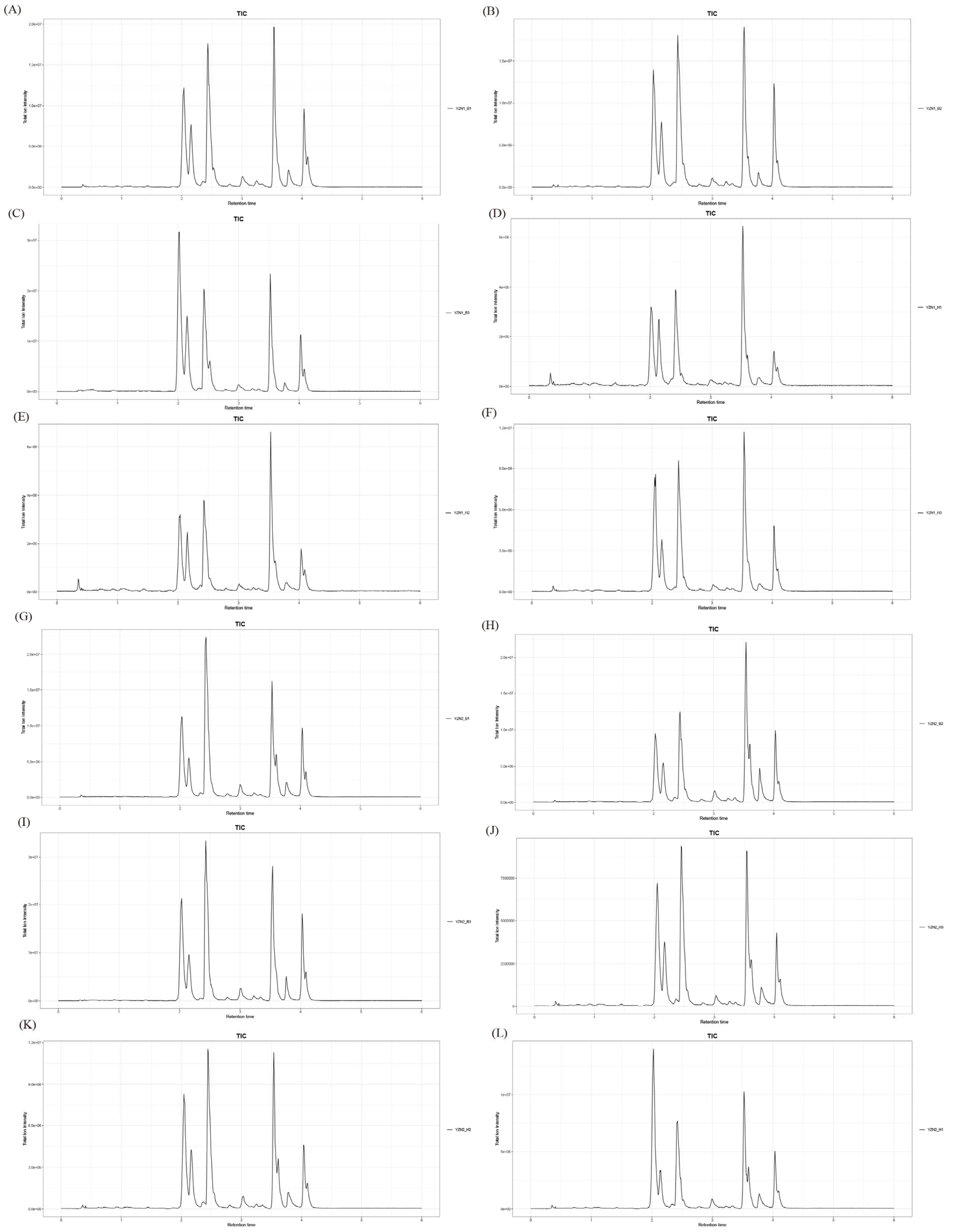

Figure S1. Total ion chromatogram (TIC) plots of the 12 samples. (**A**) YZN1_B1; (**B**) YZN1_B2; (**C**) YZN1_B3; (**D**) YZN1_H1; (**E**) YZN1_H2; (**F**) YZN1_H3; (**G**) YZN2_B1; (**H**) YZN2_B2; (I) YZN2_B3; (**J**) YZN2_H1; (**K**) YZN2_H2; (**L**) YZN2_H3.
