# Supplementary material for: Metabolites Associated with the Main Nutrients in Two Varieties of Purple Rice Processed to Polished Rice
Source: Metabolites. 2022 Dec 20;13(1):7. doi: 10.3390/metabo13010007 (PMC9867293; doi:10.3390/metabo13010007)
Supplement: Supplementary file 1 [file metabolites-13-00007-s001.zip › Table S1.pdf]

Table S1. Linear equations for the 22 amino acids.

Name	Retention Time (min)	Limit of Quantitation (μmol L^{-1})	Standard Curves	Correlation Coefficient	RSD (%)
Ala*	3.06	0.20–200.00	y = 15021.67602x + 1114.86403	0.9941	4.58
Arg*	4.05	0.10–100.00	y = 1.71579e5x + 8732.17518	0.9932	7.51
Asn*	3.63	0.10–200.00	y = 10174.75134x + 157.32107	0.9941	4.32
Asp*	3.7	1.00–200.00	y = 9770.16135x + 3639.59203	0.9936	10.34
Cys-Cys*	4.44	1.00–50.00	y = 7864.19933x + 428.75388	0.9923	6
Gln*	3.5	0.20–200.00	y = 6.90956e4x - 3922.11721	0.9931	1.03
Glu*	3.53	0.50–200.00	y = 4.48104e4x + 6773.17141	0.9925	2.2
Gly*	3.25	1.00–200.00	y = 388.88034x + 558.13920	0.9902	4.41
His*	4.1	0.10–200.00	y = 3.13333e5x + 13058.78404	0.9903	3.58
Ile*	2.08	0.05–100.00	y = 3.71350e5x + 6255.03125	0.997	4.11
Cys*	2.72	1.00–100.00	y = 3270.36842x - 66.55676	0.9919	9.09
Leu*	2.18	0.05–200.00	y = 5.07006e5x + 330.38398	0.9959	2.02
Hyp*	3.09	0.20–200.00	y = 1.14593e5x - 78.65688	0.9908	1.35
Trp*	2.07	0.10–100.00	y = 3.05543e5x - 1609.02851	0.9954	0.96
Lys*	4.11	0.10–100.00	y = 1.03898e5x + 4624.88018	0.9947	6.64
Met*	2.38	0.10–200.00	y = 1.36250e5x + 2689.83467	0.9924	3.48
Phe*	2.05	0.02–50.00	y = 1.01293e6x + 5817.99176	0.9976	0.13
Pro*	2.45	0.02–200.00	y = 1.05969e6x + 15316.58513	0.994	2.59
Ser*	3.55	5.00–200.00	y = 10132.08262x + 21382.62182	0.9903	0.68
Thr*	3.26	0.20–200.00	y = 3.27639e4x + 729.62962	0.9981	3.04
Tyr*	2.54	0.20–20.00	y = 1.22910e5 x + 9822.72634	0.9906	1.77
Val*	2.45	0.20–200.00	y = 4.84631e4 x + 1579.34431	0.9948	2.66

* - all concentrations are expressed in μmol L^{-1}.
