# Supplementary material for: Metabolites Associated with the Main Nutrients in Two Varieties of Purple Rice Processed to Polished Rice
Source: Metabolites. 2022 Dec 20;13(1):7. doi: 10.3390/metabo13010007 (PMC9867293; doi:10.3390/metabo13010007)
Supplement: Supplementary file 1 [file metabolites-13-00007-s001.zip › Table S2.pdf]

Table S2. Linear equations for ATP.

**Name**	**Retention Time (min)**	**limit of Detection (μg mL^{-1})**	**Limit of Quantitation (μg mL^{-1})**	**Standard Curves**	**Correlation Coefficient**
ATP	6.959	0.242	0.807	y = 6.1056x–0.1847	0.9999
